# Altered Passive Eruption among Patients Visiting Dental Outpatient Department in a Tertiary Care Center: A Descriptive Cross-sectional Study

**DOI:** 10.31729/jnma.8566

**Published:** 2024-05-31

**Authors:** Simant Lamichhane, Manoj Humagain, Sachita Thapa, Sita Bhusal, Arjun Hari Rijal, Prakriti Rupakhety, Rakesh Ranjan

**Affiliations:** 1 Department of Periodontics, Kathmandu University School of Medical Sciences, Kavrepalanchowk, Nepal; 2 Chandragiri Dental Home and Implant Centre Pvt. Ltd., Bhaktapur, Nepal

**Keywords:** *biotype*, *dental esthetics*, *prevalence*, *smile*, *tooth eruption*

## Abstract

**Introduction:**

The eruption of teeth is considered to be a continuous phenomenon. Two types of eruption are possible namely, active and passive eruption. Failure in passive eruption (by the apical movement of gingiva from the enamel surface) generally results in a clinical condition known as altered passive eruption. It can result in the shortened crown height of a tooth and an esthetically unpleasant situation i.e., excessive gingival display or gummy smile. The main motto of this study was to find out the prevalence of altered passive eruption and associated gingival biotypes in adult patients visiting for dental treatment in tertiary centers along with strategically placed outreach centers.

**Methods:**

A descriptive cross-sectional study was done in the Department of Dentistry, Dhulikhel Hospital, and four outreach centers of the same hospital. The study was conducted from October 2022 to April 2023 after obtaining the ethical approval. Convenience sampling was done. Point estimate and 95% Confidence Interval were calculated.

**Results:**

Among 165 patients, the prevalence of altered passive eruption was 21 (12.72%) (7.62-17.78 at 95% Confidence Interval). Furthermore, among 21 patients, the altered passive eruption was seen in the thick biotype patients in 16 (76.19%) and thin biotype patients in 5 (23.81%).

**Conclusions:**

The prevalence of altered passive eruption appeared to be equivalent when compared with the previous studies.

## INTRODUCTION

There is a movement of teeth throughout one's life. The initial movement of teeth towards the occlusal plane is known as active eruption whereas passive eruption results from the apical migration of gingiva.^1^ Failure of the dentogingival unit to move apically from the enamel is termed an altered passive eruption. Altered passive eruption (APE) has a significant impact on dentition. It commonly results in teeth with short clinical crowns and an unpleasant condition called excessive gingival display (EGD) or "gummy smile".^2^

The prevalence of altered passive eruption was around 12% in a study done by Volchansky and Cleaton-Johnes in 1974.^3^ There haven't been any studies conducted in the Nepalese population related to the altered passive eruption.

Thus, this study was aimed to find out the prevalence of the altered passive eruption among patients visiting the dental outpatient department (OPD) of a tertiary care center.

## METHODS

A descriptive cross-sectional study was conducted on the Dental Department of Dhulikhel Hospital and four other outreach centers of Dhulikhel Hospital in Dolakha (Dolakha Hospital), Kirnetar (Kirnetar Health Centre), Melamchi (Melamchi Primary Health Care Centre), and Bahunepati (Bahunepati Health Centre) from 20th October 2022 to 19th April 2023. The outreach centres were selected based on the availability of a well-equipped dental setup for conducting the designed study. The ethical approval for conducting this research was obtained from the institutional review committee of Kathmandu University School of Medical Sciences with approval number (Reference number- KUSMS number. 163/22). Informed consent was obtained from the patients included in the study. Convenience sampling was done and the sample was calculated using the following formula:


$$\begin{array}{ll} \text{n} & ={\text{Z}}^{2}\times \frac{\text{p}\times \text{q}}{{\text{e}}^{\text{2}}}\\ & =1.96^{2}\times \frac{0.121\times 0.879}{0.05^{2}}\\ & =163.44 \end{array}$$

Where,

n= minimum required sample size
Z= 1.96 at 95% Confidence interval (CI)
p= prevalence of altered passive eruption (12.1%) taken from a previous study^3^
q= 1-p
e= margin of error, 5%

The calculated sample size was 163.44. However, the total sample size taken was 165.

Informed consent was obtained from all patients after providing the information sheet regarding the study. The inclusion criteria for the current study were systemically healthy subjects of age at least 19 years of age at the time of the examination, alteration of crown height due to trauma, attrition, or restoration, no missing maxillary anterior teeth, no evidence of attachment loss, gingival overgrowth, and related conditions and cementoenamel junction (CEJ) detectable with a periodontal probe. Furthermore, the exclusion criteria set for the presented study included pregnant and lactating mothers, history of medications, smokers, and history of periodontal surgery.^4^

Clinical parameters i.e., presence or absence of altered passive eruption, gingival biotype, and excessive gingival display will be assessed by the same calibrated examiner at the mid-buccal site of the six maxillary anterior teeth. Clinical diagnosis was made using the same tool i.e., University of North Carolina 15 probe (Hu-Friedy Co., USA), mouth mirror and a calibrated stainless-steel scale in all the centers. A diagnosis of APE is determined when the distance from the gingival margin to CEJ exceeds 2 mm in ≥ 2 maxillary anterior teeth.^4^ Gingival biotypes will be assessed by the probe translucency method as thin (probe visible ≤1 mm) or thick (probe not visible >1 mm) biotypes.^5^ The excessive gingival display was said to be present when gingival exposure is >2 mm during the dynamic smile of the patient.^6^

All the data were entered in a Microsoft Excel 2010 and analyzed using IBM (International Business Machines) SPSS (Statistical Package for the Social Sciences) for windows. Point estimate and 95% CI were calculated.

## RESULTS

A total of 165 individuals were recruited in the current study. Among 165 total individuals seeking dental treatment, the prevalence of altered passive eruption was 21 (12.72%) (7.62-17.78, 95% CI). The mean age group of the participants was 29.27±9.33 years. The female-to-male ratio was around 2.5: 1 (Table 1). Excessive gingival display on the other hand was present in 17 (81%) concurrently. Excessive gingival display when present, the average value was found to be 4.85mm. APE in thick biotype was seen in 16 (76.19%) cases (Table 1).

**Table 1:** Underlying conditions of APE (n= 21).

Demographic data	n (%)
Male	6 (28.57)
Female	15 (71.53)
**Gingival biotype**	
APE with thick biotype	16 (76.19)
APE with thin biotype	5 (23.80)

## DISCUSSION

Altered passive eruption is a common clinical scenario encountered in dental practice. It has been sometimes referred to as negative gingival recession which indicates the coronal position of marginal gingiva.^7^ There are four stages of passive eruption namely I-IV as per Gargiulo et al.^8^ Stage I is where we can appreciate negative recession >2 mm whereas stage IV is considered pathological with gingival recession and loss of attachment.^8^ The prevalence of altered passive eruption in our study i.e. 12.72% concurred with previous studies done which was reported to be around 10% to 12.1%.^3,9^ The lower prevalence of APE found in the present study might be due to the selection of all dental patients. APE would have been greater if we had specifically included patients visiting the orthodontic department for treatment of gummy smile which was found around 42.1% and in a genetically similar familial trait (n%= 65).^4,10^ APE can be classified into two types: Type 1- where there is an excessive amount of keratinized gingiva and the mucogingival junction is apical to the crest of alveolar bone, and Type 2- where the mucogingival junction is shifted coronally and is apparently at the level of the alveolar crest with narrow keratinized tissue as per Coslet et al.^11^ The given classification was not evaluated and fell out of the scope of this current study. Recently, a proposed modified classification was put forward in 2017 by Zangrando et al but moreover resembles the original Coslet et al. classification system.^12^

APE usually results in unpleasant situations i.e., excessive gingival display which might result in gingivitis as well. The mean gingival exposure as shown by a study done in the Nepalese population was 4.23±1.91 mm which is comparable to our result of an average of 4.85mm.^13^ Excessive gingiva display of 2 mm is perceived as unesthetic by orthodontists and 4mm by laypersons.^14^ Thus, the amount of display can be perceived by all of our patients whoever was presented with this clinical situation.

Both the APE and EGD were found to be associated more with the thick gingival biotype in our study which was also seen in a study done by Nart et al in 2014.^4^ A simple conventional method of detection of the cementoenamel junction by the conventional periodontal probe is enough to reach into diagnosis. But, in the areas where CEJ is obliterated like in cases of cervical abrasion, it is quite difficult to diagnose.^7^

One of the main causes of APE is excessive gingival display and management protocol is with simple gingivectomy or gingivectomy with osseous reduction.^15^ The crown lengthening procedure, where there is APE along with thick gingival biotype is suggested to go with the osseous reduction technique as most often, the alveolar crest is slightly coronal to its normal position and there is a tendency of higher tissue rebound in thick tissues compared to thinner ones.^16^

The current study tried to involve different population groups by incorporating different outreach centers of Dhulikhel Hospital. However, it can't be generalized to the overall population of Nepal. Prevalence would also have been affected by selection bias as all general populations were not taken. As only a cross-sectional descriptive study was designed which limited us to finding out the causal relationship of APE with other parameters. Since this study was conducted in limited districts of Nepal, it is not representative of all the communities in the country, making generalisability limited. A wider survey including sections from various parts of Nepal would give a clearer picture.

## CONCLUSIONS

The prevalence of altered passive eruption appeared to be equivalent when compared with the previous studies. The cases were more prevalent in female subjects and in subjects with a thick gingival biotype.
